# Hypothyroidism in Pancreatic Cancer: Role of Exogenous Thyroid Hormone in Tumor Invasion—Preliminary Observations

**DOI:** 10.1155/2016/2454989

**Published:** 2016-03-31

**Authors:** Konrad Sarosiek, Ankit V. Gandhi, Shivam Saxena, Christopher Y. Kang, Galina I. Chipitsyna, Charles J. Yeo, Hwyda A. Arafat

**Affiliations:** ^1^Departments of Surgery, Jefferson Pancreatic, Biliary and Related Cancer Center, Thomas Jefferson University, Philadelphia, PA 19107, USA; ^2^Department of Biomedical Sciences, University of New England, Biddeford, ME 04005, USA

## Abstract

According to the epidemiological studies, about 4.4% of American general elderly population has a pronounced hypothyroidism and relies on thyroid hormone supplements daily. The prevalence of hypothyroidism in our patients with pancreatic cancer was much higher, 14.1%. A retrospective analysis was performed on patients who underwent pancreaticoduodenectomy (Whipple procedure) or distal pancreatectomy and splenectomy (DPS) at Thomas Jefferson University Hospital, Philadelphia, from 2005 to 2012. The diagnosis of hypothyroidism was correlated with clinicopathologic parameters including tumor stage, grade, and survival. To further understand how thyroid hormone affects pancreatic cancer behavior, functional studies including wound-induced cell migration, proliferation, and invasion were performed on pancreatic cancer cell lines, MiaPaCa-2 and AsPC-1. We found that hypothyroid patients taking exogenous thyroid hormone were more than three times likely to have perineural invasion, and about twice as likely to have higher T stage, nodal spread, and overall poorer prognostic stage (*P* < 0.05). Pancreatic cancer cell line studies demonstrated that exogenous thyroid hormone treatment increased cell proliferation, migration, and invasion (*P* < 0.05). We conclude that exogenous thyroid hormone may contribute to the progression of pancreatic cancer.

## 1. Introduction

Invasive pancreatic cancer is the fourth leading cause of cancer death in the United States. Most patients with pancreatic cancer have a dismal prognosis and a median survival rate of less than 6 months [1, 2]. At the time of diagnosis, the disease is often discovered to be in its late stages, as more than 85% of patients have tumors that have metastasized [2]. Currently, surgery remains one of the few options to decrease pancreatic cancer mortality. Despite many advances in cancer biology over the past years, pancreatic cancer remains an elusive disease process that requires further studies to understand its molecular biology and investigate possible therapeutic targets.

Thyroid hormones (T_3_ and T_4_) are steroid hormones that regulate body growth, brain maturation, and metabolism. Although the major product of the thyroid is T_4_, most of it is converted to more biologically active T_3_ that binds to nuclear thyroid receptors and modulates the expression of proteins traditionally known to increase basal metabolic rate and enhance growth [3]. Disorders of the thyroid that result in either a deficiency or excess of thyroid hormones are extremely common and can have various effects on the human body. According to the NHANES national 1999–2002 survey, the prevalence of hypothyroidism in the general US population was 3.7% [4]. Of note, the prevalence of thyroid disorders increases with age (up to 4.4% for 60 years and older) and is consistent with females having higher rates of hypothyroidism than men [5–8].

Due to the established effect of thyroid hormone on growth and development, many have hypothesized a connection between thyroid hormone and cancer. One of the first reports linking these two comes from a 1976 article that examines the relationship between supplemental thyroid hormone intake and breast cancer. In a study with 5,000 female patients, it was calculated that the rate of breast cancer in patients taking thyroid supplements for hypothyroidism was 12.1% versus 6.2% in a control group [9]. Since then, many studies have sparked a debate about a relationship between hypothyroidism and malignancy. A search of the literature reveals that hypothyroidism may be a risk factor for respiratory, colon, breast, and liver cancer [10–14]. Cell line experiments in breast and prostate cancer corroborate these findings by demonstrating that treatment with T_3_ enhances cellular proliferation [15, 16].

In this study, a retrospective analysis was performed on patients who underwent pancreaticoduodenectomy (Whipple procedure) or distal pancreatectomy and splenectomy (DPS) at Thomas Jefferson University Hospital, Philadelphia, from 2005 to 2012. The diagnosis of hypothyroidism was correlated with clinicopathologic parameters including tumor stage, grade, and survival. To further understand how thyroid hormone affects pancreatic cancer behavior, functional studies including wound-induced cell migration, proliferation, and invasion were performed on pancreatic cancer cell lines, MiaPaCa-2 and AsPC-1.

## 2. Materials and Methods

### 2.1. Data Collection

For this cross-sectional study, a database search was conducted for patients who underwent pancreaticoduodenectomy (Whipple procedure) or distal pancreatectomy and splenectomy (DPS) at Thomas Jefferson University Hospital, Philadelphia, PA, from 2005 to 2012. The eligibility criteria consisted of patients with a diagnosis of invasive pancreatic cancer confirmed by biopsy. Exclusion criteria consisted of patients with a history of noninvasive, benign pancreatic pathology or incomplete medical history. Data collection included patient sex, age, body mass index (BMI), medical history, medications, surgical information, survival, tumor staging, and differentiation by hypothyroid status. The TNM staging system as outlined by the American Joint Committee on Cancer (AJCC) was used to define pancreatic lesions. Patients were defined to be hypothyroid if they had a positive medical hypothyroidism and were taking synthetic or desiccated thyroid hormone. The Institutional Review Board of Thomas Jefferson University Hospital, Philadelphia, PA, approved this study.

MiaPaCa-2 (ATCC CRL-1420) and AsPC-1 (ATCC CRL-1682) were purchased from ATCC. MTT cell growth, migration, and transwell invasion assays were performed as previously described [17].

### 2.2. Statistical Analyses

Descriptive statistics were calculated on patient clinicopathological features. Differences in gender, smoking status, venous-lymphatic invasion, perineural invasion, T stage, N stage, prognostic stage, and differentiation by hypothyroid status were determined by Chi-square test. Differences in age and BMI were determined by unpaired Student's *t*-test. Survival analysis was performed using Kaplan-Meier curves and Mantel-Cox log rank test, where survival was defined as time between date of surgery and date of death or of last follow-up. All functional experiments were performed 3 to 5 times. Functional studies were analyzed for statistical significance by Student's *t*-test analysis, or two-way ANOVA. Data are presented as mean ± SEM. All tests of significance were two-sided with an alpha value of 0.05. Analyses were performed with the assistance of a computer program (Prism 6.0, GraphPad Software, Inc., La Jolla, CA).

## 3. Results

### 3.1. Patient Characteristics

An overview of the clinical patient data is summarized in Table 1. Of 504 patients in the database, 71 patients were found to be hypothyroid (of males, 7.7% and, of females, 20.8% were hypothyroid). As expected, the hypothyroid group had a significantly greater proportion of females than males (*P* < 0.001). The age of patients in the hypothyroid group (67.8) was similar to the age of patients in the euthyroid group (64.6). BMI was not significantly different in the hypothyroid group when compared to the euthyroid group (27.9 versus 26.3, resp.). Lastly, there was no significant difference in smoking status between two groups.

### 3.2. Pancreatic Pathology

Pancreatic tissue specimens stratified by pathology are shown in Table 2. Majority of the biopsies (85%) were invasive ductal adenocarcinoma. Second most common pathology was invasive IPMN (6%), followed by endocrine, papillary, acinar cell, and mucinous cancers.

### 3.3. Clinicopathological Parameters by Hypothyroid Status

**Figure 1:**
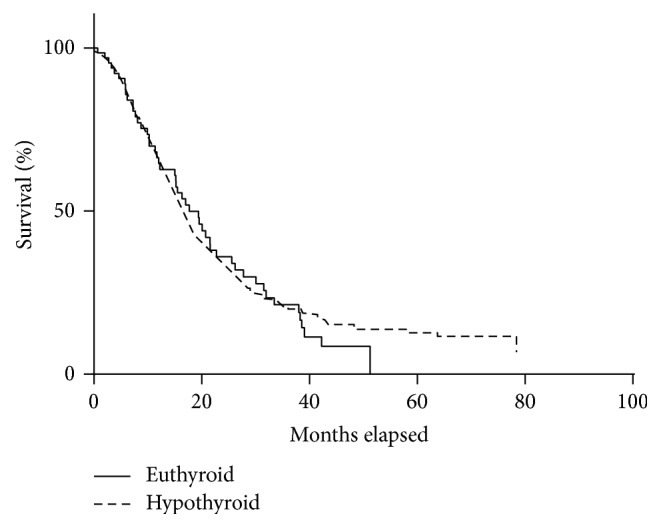
Kaplan-Meier curve comparing hypothyroid and euthyroid patients. There was no difference in survival. *P* = 0.742.

As shown in Table 3, there were no differences in survival (Figure 1), venous-lymphatic invasion, and differentiation between hypothyroid and euthyroid patients. Compared to euthyroid patients, hypothyroid patients taking exogenous thyroid hormone were more than three times likely to have perineural invasion and about twice as likely to have a higher T stage, nodal spread, and overall poorer prognostic stage.

### 3.4. T_3_ Increases Cell Proliferation, Migration, and Invasion

**Figure 2:**
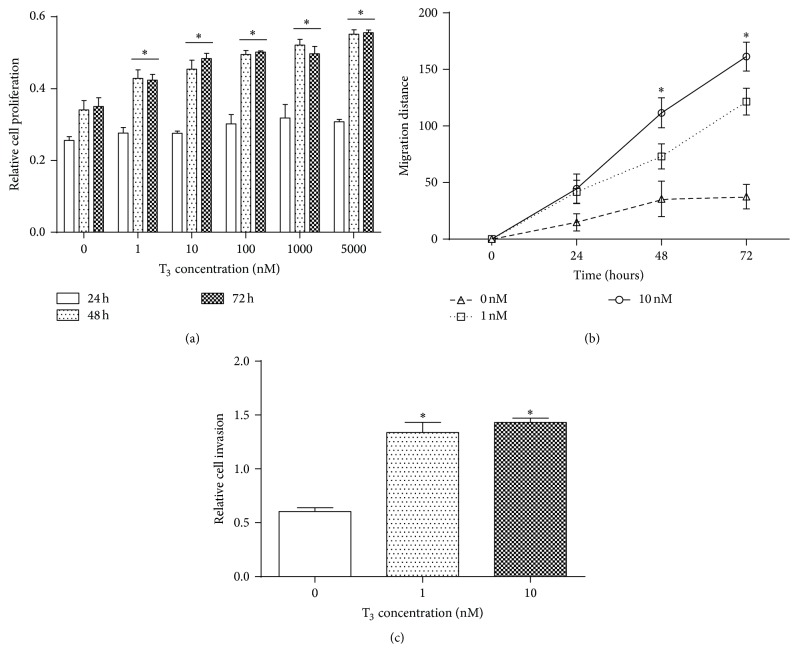
Effects of exogenous thyroid hormone treatment on PDAC cells proliferation, migration, and invasion (*P* < 0.05). (a) Proliferation assay of MiaPaCa-2 cells incubated with T_3_. Proliferation was increased at all concentrations after 48 and 72 hours. ^*∗*^ *P* < 0.05. (b) Migration assay of MiaPaCa-2 cells incubated with T_3_. Cell migration was increased after 48 and 72 hours. ^*∗*^ *P* < 0.05. (c) Transwell infiltration assay of MiaPaCa-2 cells incubated with T_3_. Cell invasion was increased by 1 and 10 nM T_3_. ^*∗*^ *P* < 0.05.

To evaluate whether T_3_ was associated with cell viability, MiaPaCa-2 cells were treated with T_3_ (0–5000 nM) and quantified via the MTT assay (Figure 2(a)). The addition of T_3_ significantly (*P* < 0.05) increased cell proliferation after 48 and 72 hours across all concentrations of T_3_. Additionally, MiaPaCa-2 cells were treated with T_3_ to evaluate its role in cell migration (Figure 2(b)). T_3_ significantly (*P* < 0.05) increased cell migration at 48- and 72-hour time points at 1 and 10 nM of T_3_ compared to the control. Lastly, the role of exogenous thyroid hormone in invasion was evaluated via transwell infiltration assay. Cells were treated with T_3_ (0–10 nM), and the extent of invasion was quantified via MTT assay (Figure 2(c)). Adding T_3_ significantly (*P* < 0.05) increased cell invasion. Similar data (*P* < 0.05) were obtained for AsPC-1 cells (data not shown).

## 4. Discussion

The objective of this study was to evaluate the prevalence of hypothyroidism and thyroid hormone supplementation in patients with pancreatic cancer and to correlate hypothyroidism diagnosis with various clinicopathologic parameters. Furthermore, functional studies were performed on MiaPaCa-2 and AsPC-1 pancreatic cancer cell lines to study how exogenous thyroid hormone influences cell behavior. To our knowledge, this is the first study to suggest a higher prevalence of thyroid hormone supplementation in patients with pancreatic cancer and to demonstrate the proliferative effects of T_3_ in pancreatic cancer cell lines.

The association between hypothyroidism and neoplasia remains controversial. Despite conflicting reports in the literature, studies have shown that hypothyroidism may correlate with many cancers including respiratory, colon, breast, and liver cancer [10–14]. Some studies even suggest that a diagnosis of hypothyroidism may result in poor response to therapy in patients with breast cancer [18]. Other studies argue that high levels of thyroid hormones induce cancer cell proliferation while low levels slow disease progress [19]. A number of prospective case-control studies have indicated that subclinical hyperthyroidism increases risk of certain solid tumors and that spontaneous hypothyroidism may delay onset or reduce aggressiveness of cancers [20–22]. A controlled prospective trial of induced hypothyroidism beneficially affected the course of glioblastoma [20].

In our study, the prevalence of patients with hypothyroidism treated with medication was 14.1% (7.7% in males, 20.8% in females). This percentage is much higher than the prevalence of overt hypothyroidism reported in the elderly (4.4%) and is consistent with females having higher rates of hypothyroidism than men [4–8].

Metastasis is one of the most significant predictors of mortality in patients with pancreatic cancer. When comparing hypothyroid and euthyroid patients with pancreatic cancer, hypothyroid patients on thyroid hormone supplementation were found to have significantly (*P* < 0.05) higher rates of nodal spread and a T stage of T3 or higher, signifying that the tumor has already extended beyond the walls of the pancreas. It was not surprising that these patients were more likely to have a poorer prognostic stage (OR 1.9). Interestingly, patients on thyroid hormone supplementation also had significantly higher rates of perineural invasion (OR 3.4). Perineural invasion is a poorly understood process by which cancer cells metastasize to nerves and their surrounding neural sheaths [23]. Although metastatic spread via neural invasion is often overlooked, PDAC has one of the highest rates of perineural invasion when compared to other malignancies, and it is a substantial cause of pain in pancreatic cancer patients [24]. Despite these poor prognostic factors, there was no significant difference in survival between patients taking thyroid hormone supplementation compared to patients who were not on medication. This might be due to the limitations of this study. One of the limitations was possible selection bias in the patient population. Because surgery was reserved for patients with resectable disease only, our study might not accurately capture patients with advanced diseases. Another limitation was little information available regarding the diagnosis of hypothyroidism. TSH measurement was not used to diagnose or quantify the degree of hypothyroidism. Similarly, the date and duration of hypothyroidism and thyroid hormone implementation were not available at the time of the surgery. Lastly, due to difficulties in patient follow-up after hospital discharge, the survival data were not the most current.

However, because all patients with hypothyroidism were taking exogenous thyroid medication, one may hypothesize that exogenous thyroid hormone may be responsible for increasing growth and metabolism of pancreatic cancer cells, thus responsible for promoting tumor invasion and spread to nearby structures. Functional assays that were performed demonstrated that treatment of MiaPaCa-2 and AsPC-1 cells with physiologic concentrations of thyroid hormone caused an increase in cell proliferation, migration, and invasion at 48 h and 72 h. These results are consistent with studies that demonstrate proliferative effect of T_3_ in breast cancer, prostate cancer, and hepatocellular carcinoma. It was also shown that T_3_ contributes to breast cancer cell proliferation through estrogen response elements mediated gene expression, by promoting the effects of estrogens themselves [15] or by upregulating TGF-*α* mRNA expression [25]. Murine glioma cell lines and human prostatic carcinoma cells also revealed the increased proliferation in response to physiological concentrations of both T_3_ and T_4_[16, 26, 27]. T_3_ also promotes cell proliferation and invasion in human hepatoma cell lines in cooperation with TGF-*β* [28, 29]. Thyroid hormones enhance the development of gastric cancer in rats by stimulating the proliferation of gastric cancer cells [14, 30]. Additionally, thyroid hormones act as growth factors in both papillary and follicular human thyroid cancer cell lines [31]. It was shown that both T_3_ and T_4_ caused proliferation of malignant glioma U-87 MG cells through PI3-kinase, Src kinase, and ERK1/2 signaling cascades [32]. T_3_ and T_4_ promote both tumor cell division and angiogenesis by activating mitogen-activated protein kinase (MAPK) via binding to a hormone receptor on the *α*v*β*3 integrin, overexpressed on many human myelomas and other cancer cells [33, 34]. Other in vitro studies of thyroid hormones action in cancer cells implicated many molecular targets, including TGF-*β*, hyperphosphorylation of Rb, and MAP kinase pathways [15, 16, 26, 35, 36]. Thyroid hormones have also been shown to promote angiogenesis in cancer cells by upregulating HIF-1*α* [35, 37].

## 5. Conclusions

This study demonstrates that there may be an association between thyroid hormone supplementation and pancreatic cancer invasion. Although the use of exogenous thyroid hormone may not necessarily be involved in the initial insult responsible for tumorigenesis, it may contribute to the progression of preexisting tumor. Increased perineural invasion, higher T stage, nodal spread, and advanced prognostic stage in hypothyroid patients may be due to enhanced metabolism of malignant cells via thyroid hormone supplementation. The proliferative effects of T_3_ on MiaPaCa-2 and AsPC-1 cells support this hypothesis.

We propose that spontaneous hypothyroidism might develop in cancer patients as a protection mechanism against tumor progress/spread, but thyroid hormone supplementation might abolish this action. Clinical studies have shown [38] that interventional lowering of serum-free T_4_ may be associated with extended survival in patients with some terminal cancers, and compassionate medical induction of hypothyroxinemia could be considered for patients with advanced cancers to whom other avenues of treatment are closed [38]. Thus, accumulating clinical evidence may justify new, broadly based controlled studies in cancer patients to determine the possible contribution of thyroid hormone to tumor behavior. Insights into molecular mechanism of this process might uncover possible targets which would allow thyroid hormone supplementation without promoting cancer progression.

## Figures and Tables

**Table 1:** Clinical characteristics of patients.

	Hypothyroid	Euthyroid	Total
	(*n* = 71)	(*n* = 433)	(*n* = 504)
Male, *n* (%)	20 (28.2)	239 (55.2)	259 (51.4)
Female, *n* (%)	51 (71.8)	194 (44.8)	245 (48.6)
Age, mean (SD)	67.8 (12.6)	64.6 (12.1)	65.1 (12.2)
BMI, mean (SD)	27.9 (5.6)	26.3 (5.3)	26.5 (5.4)
Smoking status, *n* (%), yes	32 (53.3)	200 (54.9)	232 (54.7)
Smoking status, *n* (%), no	28 (46.7)	164 (45.1)	192 (45.3)

**Table 2:** Pancreatic tissue specimens stratified by pathology.

Pancreatic malignancy	*n* (%)
Invasive ductal adenocarcinoma	427 (84.7)
Invasive IPMN	31 (6.2)
Endocrine	22 (4.4)
Papillary	17 (3.4)
Acinar cell	4 (0.8)
Mucinous	3 (0.6)
All invasive pancreatic pathologies	504 (100)

**Table 3:** Clinicopathologic parameters of hypothyroid and euthyroid patients with invasive PDA.

	Hypothyroid	Euthyroid	OR [95% CI]	*P* value
Median survival (months)	17.7	16.6		0.742
Venous-lymphatic invasion, *n* (%)			0.91 [0.52–1.58]	0.733
Yes	28 (48)	185 (51)		
No	30 (52)	180 (49)		
Perineural invasion, *n* (%)			3.38 [1.19–9.58]	0.012^*∗*^
Yes	62 (94)	335 (82)		
No	4 (6)	73 (18)		
T stage, *n* (%)			2.10 [1.00–4.37]	0.045^*∗*^
Low stage (T0–T2)	9	98		
High stage (T3-T4)	61	317		
Nodal status, *n* (%)			2.05 [1.12–3.75]	0.018^*∗*^
N0	15 (22)	157 (36)		
N1	54 (78)	276 (64)		
Prognostic stage, *n* (%)			1.89 [1.03–3.48]	0.037^*∗*^
Low stage (0–2A)	15 (22)	142 (34)		
High stage (2B-3)	54 (78)	270 (66)		
Differentiation, *n* (%)				0.612
Well	10 (14)	46 (12)		
Moderate	44 (64)	242 (61)		
Poor	15 (22)	105 (27)		

Hypothyroid patients were found to have higher rates of perineural invasion, nodal spread, and advanced prognostic stage.  ^*∗*^ *P* < 0.05.
